# Aberrant *GATA2* Activation in Pediatric B-Cell Acute Lymphoblastic Leukemia

**DOI:** 10.3389/fped.2021.795529

**Published:** 2022-01-11

**Authors:** Han Wang, Bowen Cui, Huiying Sun, Fang Zhang, Jianan Rao, Ronghua Wang, Shuang Zhao, Shuhong Shen, Yu Liu

**Affiliations:** ^1^Pediatric Translational Medicine Institute, Shanghai Children's Medical Center, School of Medicine, Shanghai Jiao Tong University, Shanghai, China; ^2^Key Laboratory of Pediatric Hematology & Oncology Ministry of Health, Department of Hematology & Oncology, Shanghai Children's Medical Center, School of Medicine, Shanghai Jiao Tong University, Shanghai, China

**Keywords:** *GATA2*, pediatric B-cell acute lymphoblastic leukemia, leukemogenesis, transcriptional dysregulation, cis-activation

## Abstract

*GATA2* is a transcription factor that is critical for the generation and survival of hematopoietic stem cells (HSCs). It also plays an important role in the regulation of myeloid differentiation. Accordingly, *GATA2* expression is restricted to HSCs and hematopoietic progenitors as well as early erythroid cells and megakaryocytic cells. Here we identified aberrant *GATA2* expression in B-cell acute lymphoblastic leukemia (B-ALL) by analyzing transcriptome sequencing data obtained from St. Jude Cloud. Differentially expressed genes upon *GATA2* activation showed significantly myeloid-like transcription signature. Further analysis identified several tumor-associated genes as targets of *GATA2* activation including *BAG3* and *EPOR*. In addition, the correlation between *KMT2A-USP2* fusion and *GATA2* activation not only indicates a potential trans-activating mechanism of *GATA2* but also suggests that *GATA2* is a target of KMT2A-USP2. Furthermore, by integrating whole-genome and transcriptome sequencing data, we showed that *GATA2* is also cis activated. A somatic focal deletion located in the *GATA2* neighborhood that disrupts the boundaries of topologically associating domains was identified in one B-ALL patient with *GATA2* activation. These evidences support the hypothesis that *GATA2* could be involved in leukemogenesis of B-ALL and can be transcriptionally activated through multiple mechanisms. The findings of aberrant activation of *GATA2* and its molecular function extend our understanding of transcriptional factor dysregulation in B-ALL.

## Introduction

Pediatric acute lymphoblastic leukemia (ALL) is the most common malignant disease in children (1). Although studies have shown improved cure rates, with the overall survival rate of pediatric ALL patients exceeding 90% (2), the prognosis of some leukemia subtypes remains poor. For example, only 75% of T-cell acute lymphoblastic leukemia (T-ALL) patients achieve remission (3), and patients with the B-cell acute lymphoblastic leukemia (B-ALL) subtypes of *BCR-ABL1, BCR-ABL1* like, and *KMT2A* (*MLL*) rearranged have a high risk of treatment failure and poor prognosis (4–7).

Advances in genome-wide analyses have enabled researchers to obtain a detailed view of the genetic alterations involved in the pathogenesis of ALL (8). The coordinated action of transcription factors controls early lymphoid development and leukemia formation, and many of these transcription factors have been found to be dysregulated in ALL (9). Patients with B-ALL harbor many genetic alterations in transcription factors including chromosomal rearrangements, deletions, or inactivating mutations in *DUX4, PAX5, IKZF1*, and *TCF3*, among others (10). For example, the *PAX5* P80R mutation impairs B cell differentiation and drives B lymphoid leukemogenesis (11). Patients with mutation or deletion of *IKZF1* were verified to have high levels of residual disease at day 19 and poor outcomes (12). T-ALL is also characterized by the dysregulation of core transcription factors, including *TAL1/2, LMO1/2*, and *TLX1/3*, caused by chromosomal rearrangements and copy number alterations (13, 14). Notably, 8–10% of T-ALL (14) and 7% of B-ALL (11) cases cannot be categorized into any of the currently established subtypes based on genome variation, and such cases commonly have inferior outcomes (8). Studies on novel driver transcription factor dysregulation could assist subtype classification and the development of targeted therapies for these patients with unknown drivers.

GATA family proteins are tissue-specific master transcriptional regulators, among GATAs, *GATA1, GATA2*, and *GATA3* have lineage-specific functions which are essential for normal hematopoiesis (15). *GATA1* is the main driver for the differentiation of hematopoietic progenitors into specific blood cell lineages, leading to a loss of self-renewal capacity (16). *GATA2* regulates the proliferation of hematopoietic stem cells (HSCs) and maintains the HSC pool (17). *GATA3* is pivotal for the development of T lymphocytes (15). Dysregulation and mutation of these three GATA transcription members are involved in leukemogenesis. For example, the *GATA3* expression level was linked to leukemogenesis (18), and recurrent mutations in the DNA binding domain of *GATA3* were detected in ETP-ALL patients and found to block T cell development (19). Heterozygous deletions of *GATA2*, which has been recognized as an acute myeloid leukemia (AML) predisposition gene (20), promote *EVI1*-provoked leukemic transformation (21). And high expression of *GATA2* in AML was associated with adverse prognosis (22). However, these findings were made only within the context of the specific lineage each GATA member was known to be physiologically associated with. Little is known regarding the aberrant activation of these transcription factors in other lineages.

In the present study, we identified the first time aberrant *GATA2* activation in a subset of B-ALL patients. The transcriptome sequencing (RNA-seq) datasets of 629 B-ALL patients obtained from St. Jude Cloud (23) were used to identify B-ALL patients with outlier *GATA2* expression. The effect of *GATA2* activation was further explored by differential expression analysis and transcription factor binding motif analysis, and several downstream targets of *GATA2* activation were identified. Finally, by integrating data from whole-genome sequencing, transcriptome sequencing, and 3-D genome analysis, we found that *GATA2* was potentially transcriptionally activated by multiple mechanisms, both cis and trans.

## Materials and Methods

### Gene Expression in Pediatric B-ALL and Differentially Expressed Gene Analysis

Count values generated from RNA-seq data of leukemia patients were downloaded from St. Jude Cloud (23). FPKM values were calculated from count values with an in-house perl script. Cases with *GATA2* FPKM values >3-fold standard deviation of the mean in each subtype were categorized as *GATA2* activation. Protein-coding genes with FPKM >1 in at least 30% of patients were defined as expressed genes and collected for further analysis. Batch effects introduced by different sequencing protocols (Supplementary Table 1) were corrected with DESeq2. Differentially expressed gene (DEG) analysis was performed on the expressed genes using DESeq2 (24) with subtype as a covariate. Genes with fold change >1.5 and *p*-value < 0.05 were identified as DEGs.

### Gene Set Enrichment Analysis

Gene set enrichment analysis of genes up-regulated and down-regulated upon *GATA2* activation was performed using ToppGene (25). The categories with top significance enriched in Gene Ontology (GO) terms and ToppCell Altas which points to cell type in ToppGene gene set were shown in figures.

### Transcription Binding Motif Analysis

GATA2 ChIP-seq data (GSM1600544) from the LNCaP cell line was used to calculate the distance between GATA2 peaks and the transcription start site (TSS) of the nearest gene to determine the genomic range for motif analysis. According to the results, the 200 bp sequences centered on the TSS of protein coding genes across the whole genome were analyzed with FIMO (26) for the presence of the transcription factor binding motif of the GATA family. Predictions with a *p*-value < 0.0001 were considered positive.

### Allelic Specific Expression Analysis

The heterozygous markers and RNA-seq BAM file for SJALL043839_D1 were obtained from previously published data (27). Allelic specific expression (ASE) analysis was performed with cis-X (28) following the user's instructions. The ASE of *GATA2* was calculated with in-house R script.

### Data Visualization

The protein structure of the in-frame KMT2A-USP2 fusion was visualized with ProteinPaint (29). The topologically associating domain (TAD) structure from high-through chromosome conformation capture (Hi-C) data from the Nalm6 and Kelly cell line, and the coverage plot of whole-genome sequencing (WGS) data of tumor and matched remission sample of SJALL043839 were visualized with GenomePaint (30).

### Statistical Analysis

The DEG analysis was performed with DESeq2. GATA family member expression between different lineages of leukemia was compared using Wilcoxon test. The correlation in expression between *GATA2* and potential target genes was calculated with Pearson correlation. All analysis were performed in R (version 3.5.3).

## Results

### Aberrant *GATA2* Transcription in B-Cell Acute Lymphoblastic Leukemia

**Figure 1:**
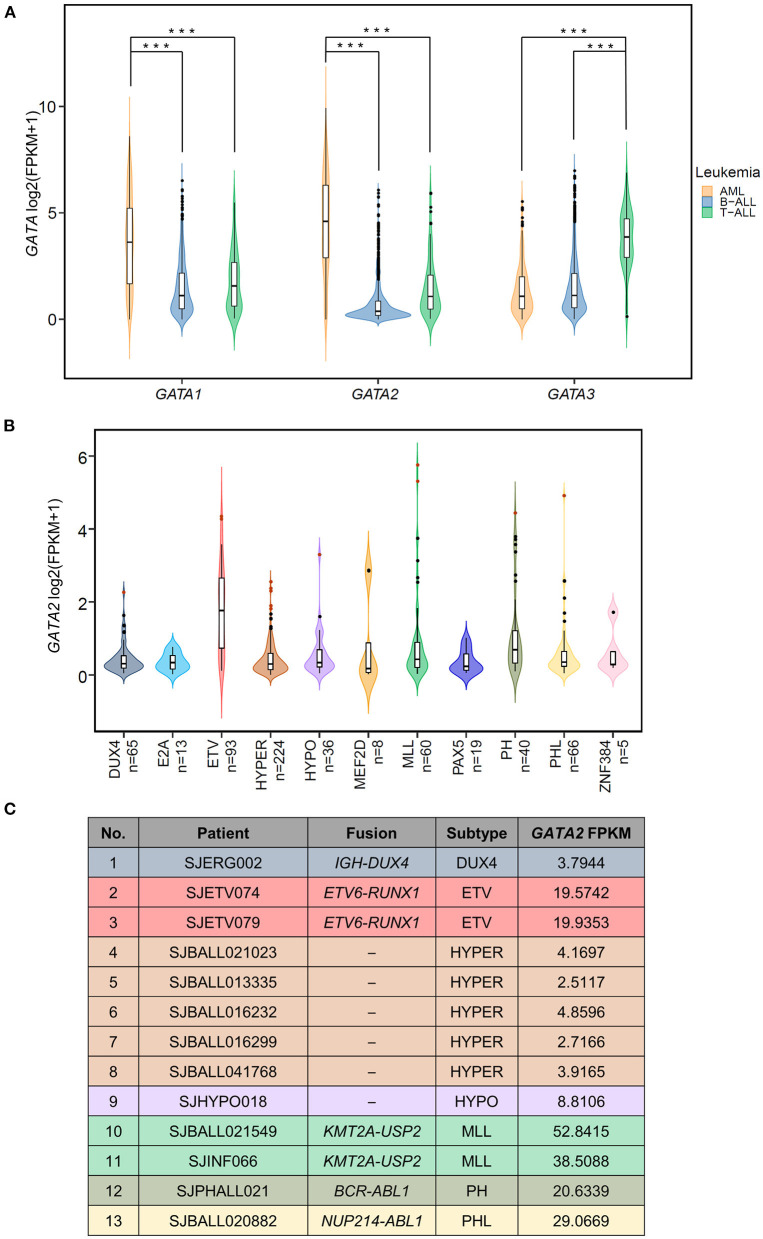
Aberrant transcription of *GATA2* in leukemia. **(A)** Violin plots showing the expression of three hematopoietic GATA transcription factors in pediatric leukemia patients; expression data are from St. Jude Cloud (AML *n* = 297, T-ALL *n* = 99, B-ALL *n* =1,248). ***p < 0.001. **(B)** The *GATA2* expression levels in 629 B-ALL patients with definitive subtype information were plotted. Thirteen *GATA2*-outlier patients were shown in red dots. **(C)** Information from 13 *GATA2*-outlier cases (i.e., sample ID, subtype, major type of fusion, and *GATA2* FPKM) is listed.

We examined the RNA expression of three hematopoietic GATA transcription factors (*GATA1, GATA2*, and *GATA3*) in a total of 1,644 pediatric leukemia patients with AML (*n* = 297), T-ALL (*n* = 99), and B-ALL (*n* = 1,248) using RNA-seq data available at St. Jude Cloud (23) (Figure 1A). Consistent with their established roles in hemopoiesis (16, 31), the three GATA transcription factors showed lineage specific patterns of transcription. *GATA1* and *GATA2* were more highly transcribed in AML as compared with B-ALL (*p*-value < 0.0001, Wilcoxon test) and T-ALL (*p*-value < 0.0001, Wilcoxon test), and *GATA3* showed the highest level of transcription in T-ALL (*p*-values < 0.0001 in comparison with AML and B-ALL, Wilcoxon test). On the other hand, transcription levels of all three GATA genes were low in B-ALL. In particular, we found that *GATA2* was not transcribed in the majority of B-ALL patients, with FPKM values <1 in 79.4% (991 out of 1,248) cases (median FPKM = 0.297). The lack of *GATA2* transcription in B-ALL was in accordance with its role in common lymphoid progenitor cells during normal hemopoiesis (32). However, it was noticeable that *GATA2* was actively transcribed in a subset of B-ALL patients (*n* = 257 with FPKM > 1).

We next asked if *GATA2* transcription was enriched in a specific B-ALL subtype. Toward this end, we checked transcription levels of *GATA2* in 629 of the 1,248 B-ALL cases mentioned above, for which definitive subtype information was available. First of all, we found that active transcription of *GATA2* was associated with *ETV6-RUNX1* rearranged B-ALL (ETV, Figure 1B, median FPKM = 2.26, *p* < 0.0001, Wilcoxon test, ETV compared with non-ETV). Secondly, aberrantly high *GATA2* transcription was observed in a subset of B-ALL patients across different subtypes. We next identified the cases with outlier *GATA2* transcription defined as *GATA2* transcription levels greater than three standard deviations of the mean in each subtype. Aberrant transcription was analyzed within each subtype to minimize the variation across different subtypes. According to this criterion, a total of 13 B-ALL cases with aberrant activation of *GATA2* were identified from 7 subtypes (Figure 1B), accounting for 2.07% of B-ALL cases. These 13 B-ALL cases included five with high hyperdiploid (HYPER), two with *KMT2A* rearrangements (MLL), two with ETV, and one each with *DUX4* rearrangement (DUX4), low hypodiploid (HYPO), *BCR-ABL1* (PH), and *BCR-ABL1*-like (PHL) (Figure 1C). In addition, no outlier *GATA2* transcription was identified for the following four subtypes: *TCF3-PBX1* (E2A), *MEF2D* rearrangements (MEF2D), *PAX5* alterations or mutations (PAX5), and *ZNF384* rearrangements (ZNF384) (Figure 1B; Supplementary Table 1). To further investigate the effect of *GATA2* activation, we selected 584 patients from seven subtypes with at least one *GATA2* outlier case identified. Among these 584 cases, 13 cases with aberrant activation of *GATA2* were classified as the *GATA2*-outlier group and the remaining cases (*n* = 571) as the *GATA2*-normal group.

### Dysregulated *GATA2* Transcription Activates Myeloid Lineage-Specific Genes Transcription in B-ALL

**Figure 2:**
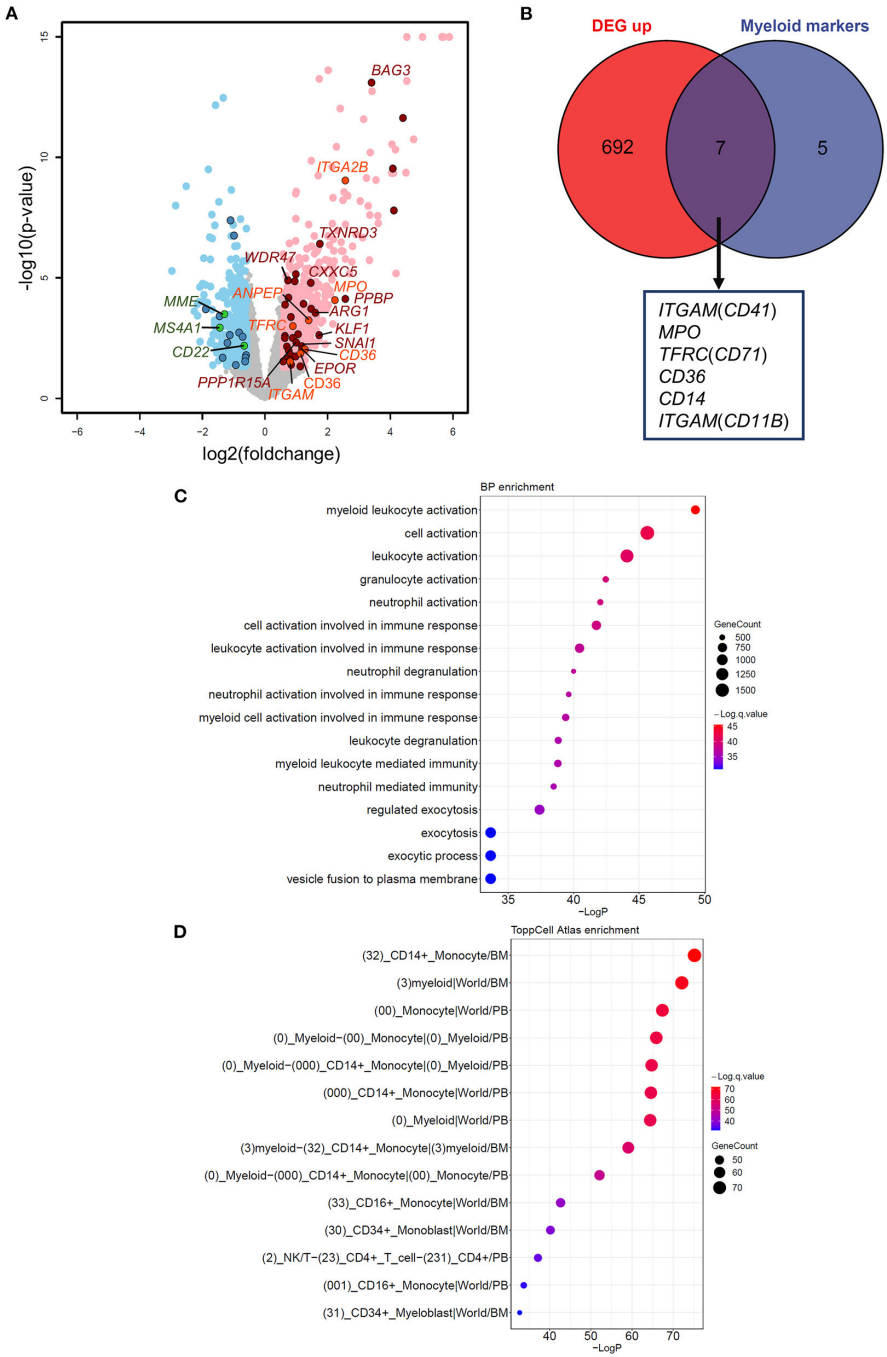
Effects of aberrant *GATA2* transcription on B-ALL. **(A)** Volcano plot illustrating the differentially expressed genes (DEGs) between *GATA2*-outlier patients and *GATA2*-normal patients. Gray dots indicate genes with no significant differences. Light red and light blue dots indicate 699 up-regulated and 451 down-regulated DEGs, respectively, in *GATA2*-outlier patients (*p*-value < 0.05 and |FC| > 1.5). DEGs predicted to be bound by *GATA2* in motif analysis are highlighted in dark red (up-regulated) and dark blue (down-regulated). DEGs overlapped with Myeloid markers and B cell markers commonly used in clinical diagnosis are highlighted in orange (up-regulated) and green (down-regulated). **(B)** Intersection between 699 up-regulated DEGs (left) and myeloid markers tested in clinical diagnosis (right). Seven out of 12 myeloid markers were upregulated in *GATA2*-outlier cases and labeled below. **(C,D)** Results of gene set enrichment analysis showing that 699 up-regulated DEGs are enriched in myelocyte-associated Biological Process (BP) and ToppCell Altas categories. The categories with a *p*-value < 10^−30^ are listed and sorted by *p*-value in reverse order. The color of each dot represents *q*-value, and the size of the dot represents the number of DEGs in each category.

To understand the impact of dysregulated *GATA2* transcription on B-ALL, we performed differential expression analysis between the *GATA2*-outlier group and the *GATA2*-normal group using DESeq2 with B-ALL subtypes as a covariate. Out of 10,505 expressed protein coding genes (FPKM >1 in >30% patients), we identified 1,150 differentially expressed genes (DEGs), including 699 up-regulated and 451 down-regulated, in *GATA2*-outlier cases (*p*-value < 0.05 and |fold change| >1.5; Figure 2A; Supplementary Table 2). We firstly checked the transcription of B cell and myeloid lineage specific markers commonly tested in clinical diagnosis in the DEG result (Supplementary Table 3). Seven out of twelve myeloid markers used in clinical test were significantly up regulated in *GATA2*-outlier cases (Fisher's exact test, *p* < 0.001) (Figures 2A,B), while none of the 12 myeloid markers were down-regulated. On the other hand, B-lineage markers were significantly down regulated (Fisher's exact test, *p* < 0.001) (Figure 2A; Supplementary Figure 1A) and none of these B-lineage markers showed up-regulated expression. We next performed gene set enrichment analyses on up-regulated and down-regulated DEGs separately using ToppGene tool (25). Consistently, the 699 up-regulated DEGs were significantly enriched in myeloid leukocyte-associated enrichment categories including both GO (terms with a *p*-value < 10^−33^ are listed in Figure 2C) and ToppCell Atlas (categories with a *p*-value < 10^−33^ are listed in Figure 2D), suggesting the activation of a myeloid lineage-related transcription circuit following *GATA2* dysregulation (15). In contrast, the 451 down-regulated DEGs were significantly enriched for the early B-lineage precursor cells stage (Supplementary Figure 1B) from which B-ALL leukemic cells originate. These observations indicated a potential switch from B-cell lymphocytes related transcription circuit to myeloid lineage related transcription circuit upon *GATA2* activation. Down-regulated DEGs were also significantly enriched for cell cycle process (Supplementary Figure 1C), implying that *GATA2* could be involved in the proliferation of blasts in *GATA2*-outlier patients.

### *GATA2* Activates Transcription of Tumor-Associated Genes in B-ALL

To investigate the targets of *GATA2* activation that are potentially involved in B-ALL, we combined genome-wide computational prediction of GATA family transcription factor binding motifs and the differential expression analysis described above. We first analyzed GATA2 chromatin immunoprecipitation sequencing (ChIP-seq) profiles of LNCaP cancer cell line published previously (33) to estimate the distance between each GATA2 binding site and its potential target gene. Only protein coding genes were included in this analysis. We found that among the GATA2 peaks with a nearby potential target gene (within 2 kb of the peak), 37.3% were located within ±100 bp of the transcription start site (TSS) of the nearby gene (Supplementary Figure 2A). This suggested that GATA2 targets downstream genes by predominantly binding to the ±100 bp of TSS region. Therefore, we scanned the TSS ±100 bp sequences of all protein coding genes across the human genome to predict GATA2 binding sites. This resulted in the identification of 313 genes with one or more GATA family binding motifs that may be direct targets of GATA factors (*p*-value < 0.0001 with FIMO, Supplementary Table 4). The predictions were confirmed by gene set enrichment analysis; the 313 genes showed significant enrichment in experimentally derived GATA binding sites from the ChIP-seq analysis in ENCODE project (34, 35) (Supplementary Figure 2B), supporting the prediction of GATA2 targets.

**Figure 3:**
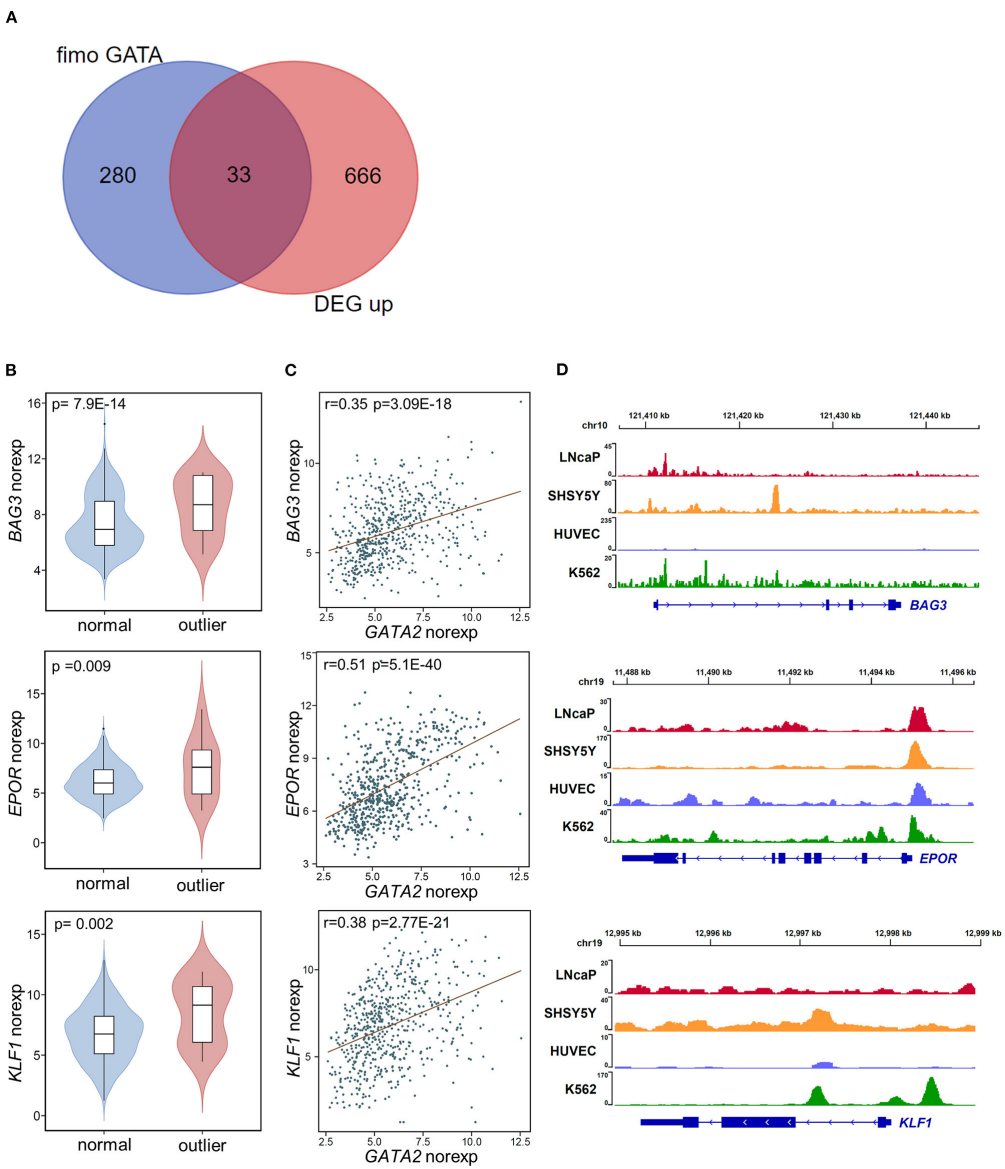
Potential targets of *GATA2* activation. **(A)** The overlap between potential *GATA2*-targeted genes predicted by FIMO (left) and up-regulated DEGs (right). **(B)** Violin plots showing the normalized expression of the potential *GATA2*-targeted genes *BAG3, EPOR*, and *KLF1* in the *GATA2*-outlier and *GATA2*-normal groups. The *p*-value from differential expression analysis is shown above each plot. **(C)** Scatter plots indicating the correlations between normalized expression levels of *GATA2* (x axis) and the potential target genes (y axis). The correlation coefficient and *p*-value were determined by Pearson correlation analysis. **(D)** Wiggle plot of GATA2 ChIP-seq data from four tumor cell lines showing that the GATA2-binding site is located near the potential target genes in **(B)**.

We next integrated the DEGs from differential expression analysis with the 313 targets of GATA factors from computational prediction. We identified 46 potential targets dysregulated by aberrant activation of *GATA2*, including 13 down-regulated DEGs (Supplementary Figure 2C) and 33 up-regulated DEGs (Figure 3A; Supplementary Table 2). We focused on the up-regulated DEGs in this analysis. Notably, there were several known tumor-associated genes (highlighted in Figure 2A) among these 33 target genes, including *BAG3, EPOR*, and *KLF1*. *BAG3*, which was reported to be an anti-apoptotic gene involved in leukemic cell survival and response to therapy (36), was among the most significant DEGs between *GATA2*-outlier and *GATA2*-normal B-ALL patients (*p*-value = 7.9 × 10^−14^; log2FC = 3.4, Figure 3B). And there was a significant positive correlation between *GATA2* and *BAG3* transcription in B-ALL (*r* = 0.35, *p*-value = 3.09e-18, Figure 3C). Furthermore, GATA2 binding was observed in ChIP-seq experiments from multiple tumor cell lines, namely LNCaP, HUVEC, SHSY5Y, and K562 (Figure 3D). Taken together, these observations support the hypothesis that *BAG3* is a target of aberrantly activated *GATA2* in B-ALL. Similar observations were made for *EPOR* and *KLF1*; both genes showed significant co-expression with *GATA2* (Figure 3C) and positive GATA2 binding observed for multiple cancer cell lines in ChIP-seq experiments (Figure 3D). *EPOR* is a well-established B-ALL-associated gene whose abnormal activation in pre-B cells increases cell survival by activating the JAK-STAT pathway (37), whereas *KLF1* is associated with poor survival in AML (38). The up-regulation of these genes by *GATA2* activation may play a tumor-promoting role in leukemia and reinforce the potential function of *GATA2* in leukemogenesis in B-ALL.

### Trans- and Cis-Activation of *GATA2* in B-ALL

**Figure 4:**
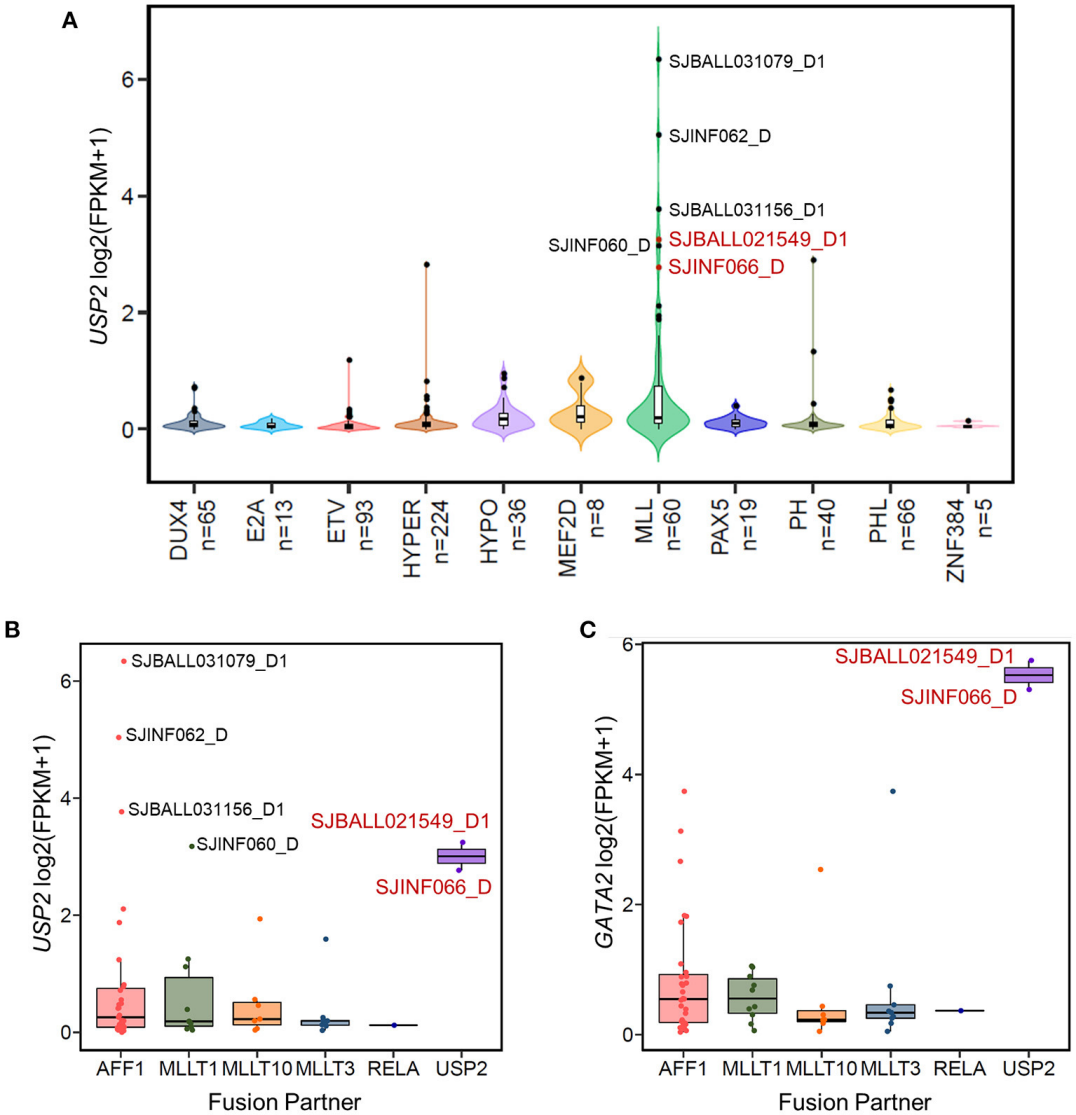
The *KMT2A-USP2* fusion activates *GATA2* in B-ALL. **(A)** The expression of *USP2* in 629 B-ALL patients across 11 subtypes. Six MLL B-ALL patients with *USP2* FPKM values >5 are labeled with the corresponding sample IDs. Two patients with the *KMT2A-USP2* fusion are highlighted in red. **(B)** Boxplot showing *USP2* transcription in patients with the MLL subtype of B-ALL. Cases were further divided into groups according to *KMT2A* fusion partner. Six *USP2*-positive MLL cases are labeled and color coded as in **(A)**. **(C)** The expression of *GATA2* across different fusion partner groups in MLL subtypes as shown in **(B)**.

In the 13 *GATA2*-outlier B-ALL patients, two were MLL subtype (SJBALL021549_D1 and SJINF066_D). Notably, we found that both patients carried the same *KMT2A-USP2* fusion. Further checking confirmed that these patients were the only two carrying this fusion in the cohort analyzed (Supplementary Table 1), indicating that *GATA2* could be trans-activated by KMT2A-USP2. *KMT2A-USP2* is a relatively rare type of *KMT2A* rearrangement and was recently reported as a recurrent fusion in pediatric B-ALL (39). In the two cases identified in this analysis, the breakpoint of the fusion was located within intron 22 of *KMT2A* (NM_005933) and intron 2 of *USP2* (NM_004205), which resulted in an in-frame fusion consisting of exons 1–21 of *KMT2A* and exons 3–13 of *USP2* (Supplementary Figure 3). The fusion protein contains the entire deubiquitination domain of USP2, indicating that USP2 might be the functional executor of this chimeric protein. Interestingly, wild-type *USP2* was not transcribed in majority of B-ALL patients (Figure 4A) and could be activated through fusion with *KMT2A* (Figure 4B), with active transcription of *USP2* occurring through hijacking of the *KMT2A* promoter (Supplementary Figure 3). In addition, we found high levels of wild-type *USP2* transcription in couple of B-ALL patients with other MLL subtype (Figures 4A,B), indicating *USP2* could also be aberrantly activated in *KMT2A* rearranged B-ALL. Notably, *GATA2* outlier expression was found in patients with the *KMT2A-USP2* fusion but not in other *KMT2A* rearranged patients with high expression of wild-type *USP2* (Figure 4C), suggesting that *GATA2* transcription was activated by the chimeric fusion protein as a whole.

**Figure 5:**
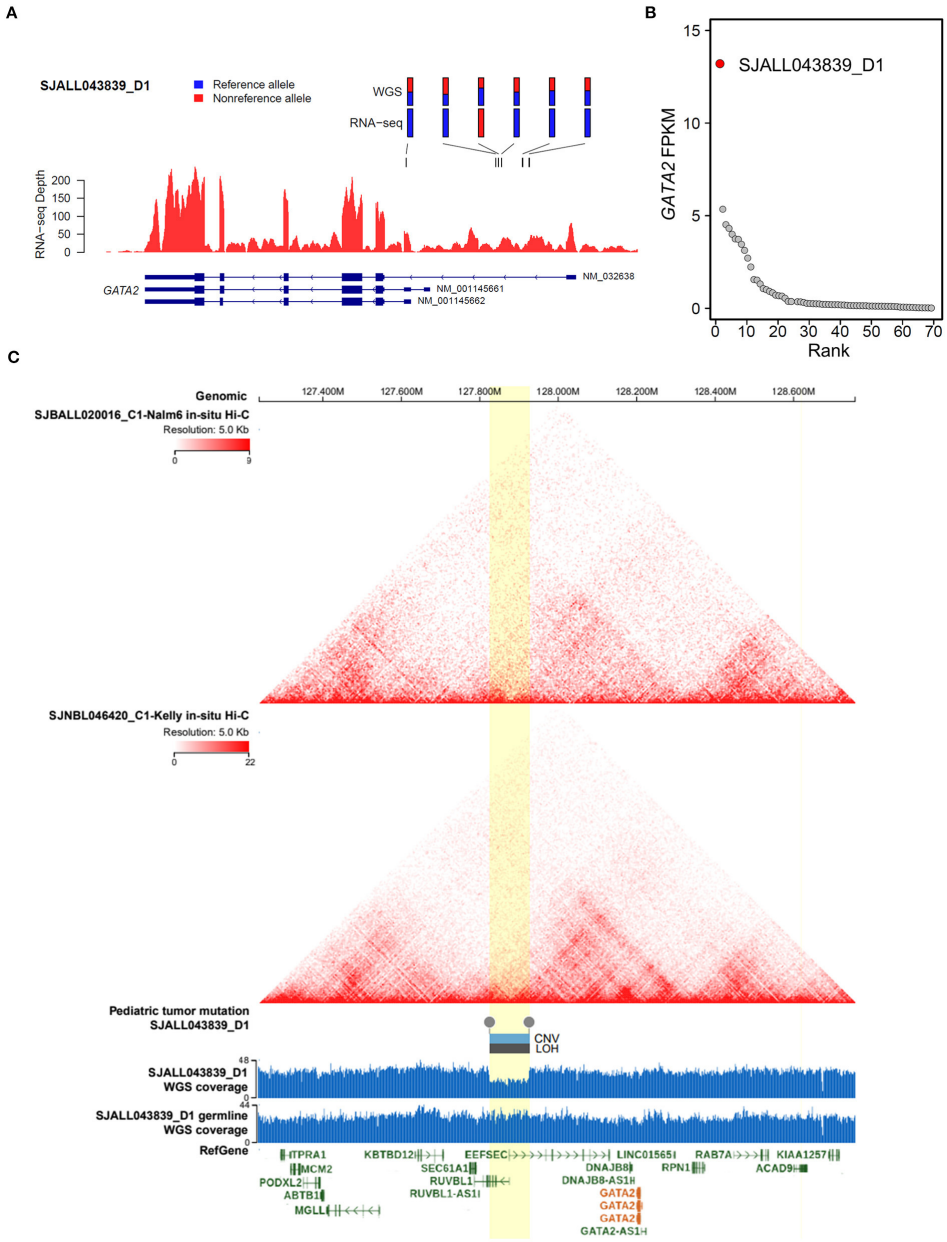
Cis-activation of *GATA2* due to a downstream focal deletion. **(A)** Allelic specific expression of *GATA2* in SJALL043839_D1 was determined by examining the mono-allelic expression of six heterozygous germline/somatic variants, which are labeled above the wiggle plot, with blue and red representing the reference and alternative alleles, respectively. **(B)** Outlier high expression (FPKM on the y axis) of *GATA2* was found in sample SJALL043839_D1 (red dot) from the Shanghai Children's Medical Center ALL cohort. **(C)** Focal deletion activating GATA2 in SJALL043839_D1. Topologically associating domains from Hi-C data of the Nalm6 and Kelly cell lines are displayed on the top; both pictures show a wild-type 3-D genome architecture at the *GATA2* locus. A somatic focal deletion was identified in the tumor cells of SJALL043839_D1; the deletion is shown at the bottom with a wiggle plot showing the coverage from whole-genome sequencing data of tumor and matched remission samples. The deletion is indicated by boxes above the wiggle plot, which represent the copy number variation (CNV, log_2_Ratio = −0.75) and the loss of heterozygosity (LOH, segment mean value = 0.32). The focal deletion and the candidate TAD boundary disrupted by the deletion are highlighted in yellow. The whole-genome sequencing and Hi-C data were visualized with GenomePaint (30) (https://genomepaint.stjude.cloud/).

Meanwhile, we were able to identify another MLL B-ALL case, SJALL043839_D1, with aberrant *GATA2* transcription from a previously published ALL cohort (27), in which both RNA-seq and whole-genome sequencing (WGS) data were available for tumor cells. By integrating WGS and RNA-seq data with cis-X (28), we found significant allele-specific expression (ASE, Figure 5A) as well as outlier expression of *GATA2* in this case compared with the remaining B-ALL patients from the same cohort (Figure 5B). Further analysis identified a somatically acquired 101 kb focal deletion located 285 kb downstream of the TSS of *GATA2* in the tumor cells (Figure 5C). Notably, *GATA2* was the only cis-activated gene in the genomic neighborhood surrounding this deletion (Supplementary Figures 4A–C), indicating that *GATA2* was the target. Furthermore, *in-situ* high-through chromosome conformation capture (Hi-C) data for both the B-ALL cell line Nalm6 and neuroblastoma cell line Kelly (Figure 5C) from GenomePaint (30) showed that the deletion overlapped with the boundary of topologically associating domain (TAD) harboring *GATA2*. Disruption of the TAD structure by the deletion might aberrantly connect the *GATA2* promoter to the active regulatory DNA elements in the adjacent TAD, thus contributing to *GATA2* dysregulation in cis.

## Discussion

The expression of transcription factors is strictly regulated during development, both spatially and temporally. Aberrant activation of transcription factors is one of the most common drivers in ALL (9, 40). *GATA2* is actively expressed in multipotent progenitors, erythroid precursors, and myeloid precursors, but not in lymphoid precursors or B-ALL (16, 41). In the present study, we first reported low expression of *GATA2* in the majority of B-ALL patients, which was consistent with the origination of this tumor from the early developmental stage of B lymphoid. On the other hand, we showed that *GATA2* was actively transcribed in a subset of B-ALL patients, especially in those with the ETV subtype (Figure 1B). The transcription of *GATA2* in ETV-subtype B-ALL patients is supported by a recent study carried out with the B-ALL cell lines REH and Nalm6, which belong to the ETV and DUX4 subtypes respectively, suggesting potential association between *GATA2* transcription and gene methylation (42). In addition, we discovered 13 B-ALL cases with aberrant *GATA2* activation from 629 B-ALL patients, indicating that *GATA2* dysregulation is a shared feature of different B-ALL subtypes. Cluster analysis based on 1150 DEGs between *GATA2*-outlier cases and *GATA2*-normal cases showed that patients could not be grouped by *GATA2* transcription level (Supplementary Figure 1D). This was partially because of the limited number of *GATA2* outlier cases included in this analysis, but it also indicated that *GATA2* activation was a secondary event in B-ALL and would need to cooperate with other driver genomic aberrations in leukemogenesis. Further investigations including more B-ALL cases with *GATA2* activation are needed to unveil detailed co-occurrence relationship between *GATA2* activation and other driver genomic aberrations. Meanwhile, the myeloid-specific gene signature observed in these cases indicated that *GATA2* activation rewires gene transcription circuits in B-ALL cells toward myeloid-like (Figures 2C,D), which is consistent with the expression of *GATA2* in early myeloid precursors (43). On the other hand, it was reported that *GATA2* deficiency restricts cell proliferation in AML cells and delays leukemogenesis in *Cbfb-Myh11* knock-in mice, supporting the notion that *GATA2* is a poor prognostic marker in pediatric AML (22). Indeed, we found that several up-regulated DEGs targeted by *GATA2* in the *GATA2*-outlier B-ALL cases were known tumor-associated genes (Figure 2A), including *BAG3* and *EPOR*. This provides further evidence for the potential tumor promoting function of *GATA2* in B-ALL.

The two MLL subtype patients (SJBALL021549_D1 and SJINF066_D) with *GATA2* activation both carried the *KMT2A*-*USP2* fusion, which is rare in B-ALL and its function has been largely unexplored. Although patient SJINF066 was an infant (9-month-old) and SJBALL021549_D1 was a 9-year-old child, the expression levels of *GATA2* in these two cases were comparable, indicating that trans-activation of *GATA2* by KMT2A-USP2 is not influenced by age but rather by the function of this fusion. Different from other common *KMT2A* rearrangements with breakpoints located in the major breakpoint cluster region (BCR), the *KMT2A*-*USP2* breakpoint appeared to be inside the minor BCR and joined the plant homeodomain (PHD) domain of KMT2A with the conserved ubiquitin carboxyl-terminal hydrolase (UCH) domain of USP2 (39). The biological function of *KMT2A-USP2* in ALL is still unclear. Available hypotheses have mainly focused on the function of USP2 including USP2-mediated deubiquitination of KMT2A and cell cycle-related cyclin proteins (39, 44). However, we noticed that high expression of wild-type *USP2* did not result in aberrant *GATA2* transcription (Figure 4C), indicating that both parts of the KMT2A-USP2 fusion protein contribute to *GATA2* activation. Considering that the *KMT2A* fusion trans-activates *HOX* genes by directly binding to *HOX* loci (45) and that histone H2A ubiquitination plays a critical function in polycomb-mediated transcriptional repression (46, 47), it is likely that USP2 is relocated to the nucleus by the KMT2A-USP2 fusion and contributes to trans-activation of *GATA2* transcription through deubiquitinating H2A and further disrupting gene silencing maintained by polycomb group complexes. Further experiments are needed to investigate the molecular mechanism. In a previously published study of a cohort of 11 pediatric B-ALL patients with the *KMT2A-USP2* fusion, three patients presented with central nervous system disease and 8 patients had positive-minimal residual disease at day 33, suggesting a poor prognosis of patients carrying this fusion (39). Our results identifying *GATA2* as a potentially novel target of the KMT2A-USP2 fusion might provide novel clues for understanding the function of this fusion.

Our data showed that in addition to the trans-activation of *GATA2* by the KMT2A-USP2 fusion, a cis-activating mechanism is also involved in *GATA2* dysregulation. ASE and high outlier expression of *GATA2* were found in one B-ALL patient with a somatic focal deletion detected downstream of *GATA2* (Figures 5A,B). Hi-C data from Nalm6 and Kelly cell lines verified that the candidate insulated boundaries were potentially disrupted by the deletion (Figure 5C). The evidence confirmed that the focal deletion altered the conserved three-dimensional genome structure thus cis-activating *GATA2* in B-ALL. This mechanism is similar to the activation of other oncogenes including *TAL1* and *LMO2* by disruption of TADs (48). Interestingly, SJALL043839_D1 is also a MLL B-ALL (*KMT2A-MLLT3*). As the *KMT2A* rearrangement was an unfavorable prognostic factor (5, 6) in ALL, further investigation of the potential interplay of aberrant *GATA2* transcription and *KMT2A* fusions might shed new light on the molecular basis of this subtype of B-ALL.

Taken together, our study revealed aberrant *GATA2* activation in B-ALL patients. Abnormal *GATA2* transcription makes B-ALL tumor cells more myeloid-like and could contribute to leukemogenesis by activating downstream target genes.

## Data Availability Statement

The RNA-seq data for the St. Jude leukemia patients (297 AML cases, 99 T-ALL cases, and 1248 B-ALL cases) were downloaded from St. Jude cloud (https://www.stjude.cloud). The GATA2 ChIP-seq data used in this study can be accessed from the gene expression omnibus under accession nos. GSM1600544, GSM935347, GSM935589, and GSM935373 for LNCaP, HUVEC, SHSY5Y, and K562 cells, respectively, with the called peaks (in BED format) available upon request. Whole-genome sequencing and RNA-seq data for SJALL043839_D1 are available in St. Jude Cloud GenomePaint (https://genomepaint.stjude.cloud/). The Hi-C data of Nalm6 and Kelly cell lines are also available from St. Jude Cloud GenomePaint.

## Author Contributions

HW, BC, and HS performed statistical analysis. FZ collected clinical information. JR, RW, and SZ analyzed the genomic data of SJALL043839_D1. SS and YL designed and supervised the study. HW and YL wrote the manuscript. All authors contributed to the article and approved the submitted version.

## Funding

This work was supported by the National Natural Science Foundation of China (31970627 to YL) and Shanghai Key Laboratory of Clinical Molecular Diagnostics for Pediatrics (20dz2260900 to YL).

## Conflict of Interest

The authors declare that the research was conducted in the absence of any commercial or financial relationships that could be construed as a potential conflict of interest.

## Publisher's Note

All claims expressed in this article are solely those of the authors and do not necessarily represent those of their affiliated organizations, or those of the publisher, the editors and the reviewers. Any product that may be evaluated in this article, or claim that may be made by its manufacturer, is not guaranteed or endorsed by the publisher.

## Supplementary Material

Supplementary Figure 1 The effect of *GATA2* activation on differentially expressed genes in B-ALL. **(A)** Intersection between down-regulated DEGs (left) and B cell markers (right). Three out of five B cell markers were down regulated in *GATA2*-outlier cases and listed below. **(B)** A total of 451 down-regulated DEGs were enriched in pre-B or pro-B lymphoid cell-related categories in the ToppCell Atlas. Significantly enriched categories (*p*-value < 10^−15^) are shown. **(C)** Gene Ontology enrichment results for 451 down-regulated DEGs; categories are associated with the cell cycle. The categories with a *p*-value < 10^−7^ are shown and sorted by *p*-value in reverse order. **(D)** Cluster analysis of *GATA2*-outlier (*n* = 13, big circles) and *GATA2*-normal samples (*n* = 571, small circles) using 1150 DEGs from differential expression analysis. The results are shown as a t-SNE plot. Each dot represents an individual B-ALL patient and the color indicates the B-ALL subtype.

Supplementary Figure 2 Prediction of *GATA2* targets. **(A)** Distribution of distances between *GATA2* peaks and the nearest TSS of a gene in the GATA2 ChIP-seq from LNCaP (GSE38391). Only peaks within the promoter region (−1000 to +1000 bp) of at least one protein coding gene were included. **(B)** The enrichment results of 313 potential GATA-targeted genes. The number of target genes in each enriched category is shown on the x-axis. The color represents the *p*-value. **(C)** Intersection of potential *GATA2* target genes predicted by FIMO (left) and DEGs down-regulated upon *GATA2* activation (right).

Supplementary Figure 3 *KMT2A-USP2* fusion in MLL B-ALL patients with outlier *GATA2* transcription. Protein diagrams were generated using ProteinPaint (29) (https://proteinpaint.stjude.org/).

Supplementary Figure 4 The transcription of genes near the deletion downstream of *GATA2*. The expression levels of *EEFSEC* **(A)**, *RUBBL1* **(B)**, and *SEC61A1* **(C)** in B-ALL patients from the Shanghai Children's Medical Center ALL cohort are shown as scatterplots. Expression level (FPKM) is plotted on the y-axis. The case with cis-activated *GATA2* (SJALL043839_D1) is highlighted in red in each panel.
