# Supplementary figures and images for: Aberrant GATA2 Activation in Pediatric B-Cell Acute Lymphoblastic Leukemia
Source: Front Pediatr. 2022 Jan 11;9:795529. doi: 10.3389/fped.2021.795529 (PMC8787225; doi:10.3389/fped.2021.795529)

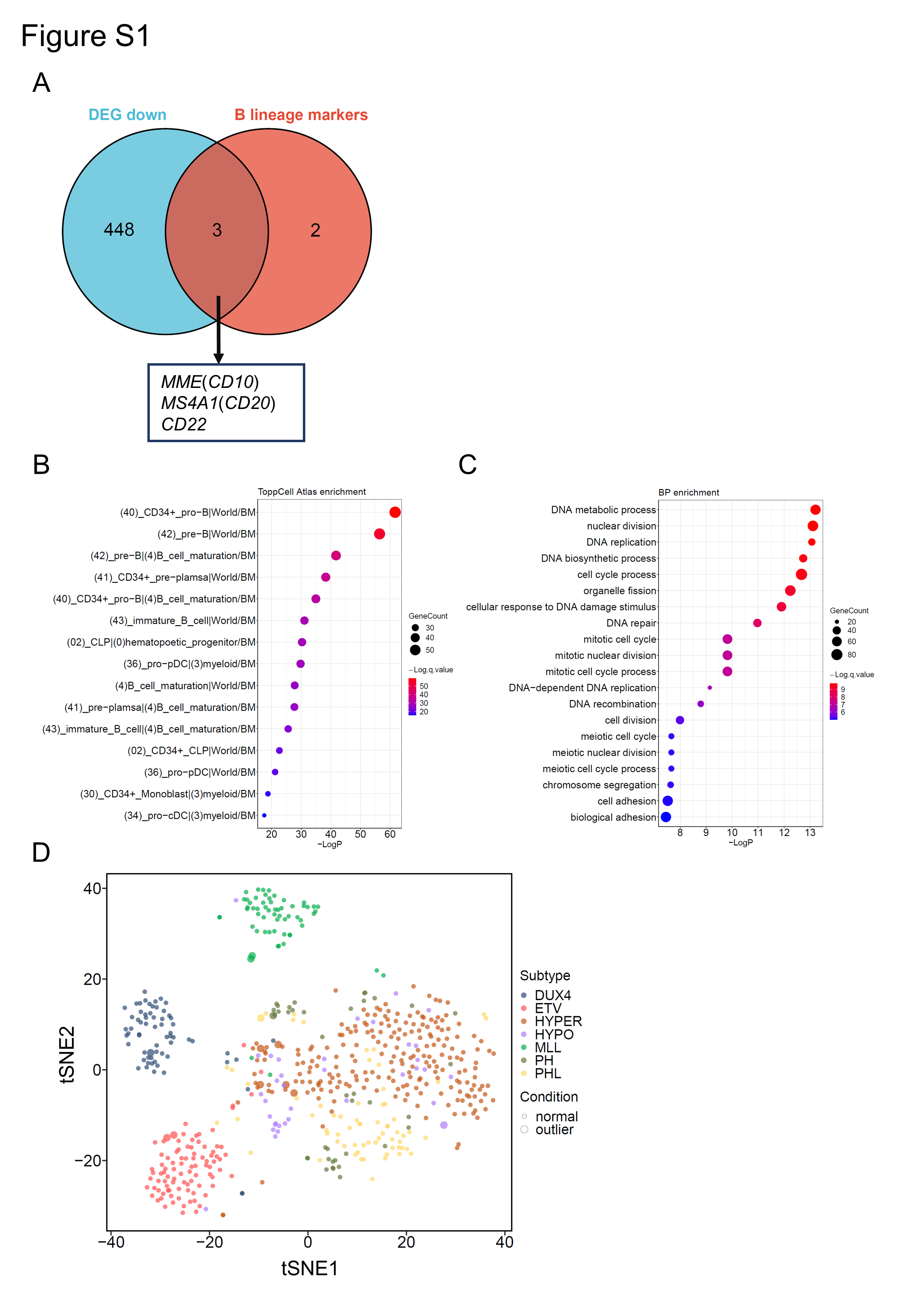

Supplement: Supplementary Figure 1 — The effect of GATA2 activation on differentially expressed genes in B-ALL. (A) Intersection between down-regulated DEGs (left) and B cell markers (right). Three out of five B cell markers were down regulated in GATA2-outlier cases and listed below. (B) A total of 451 down-regulated DEGs were enriched in pre-B or pro-B lymphoid cell-related categories in the ToppCell Atlas. Significantly enriched categories (p-value < 10−15) are shown. (C) Gene Ontology enrichment results for 451 down-regulated DEGs; categories are associated with the cell cycle. The categories with a p-value < 10−7 are shown and sorted by p-value in reverse order. (D) Cluster analysis of GATA2-outlier (n = 13, big circles) and GATA2-normal samples (n = 571, small circles) using 1150 DEGs from differential expression analysis. The results are shown as a t-SNE plot. Each dot represents an individual B-ALL patient and the color indicates the B-ALL subtype. [file Image_1.TIF]

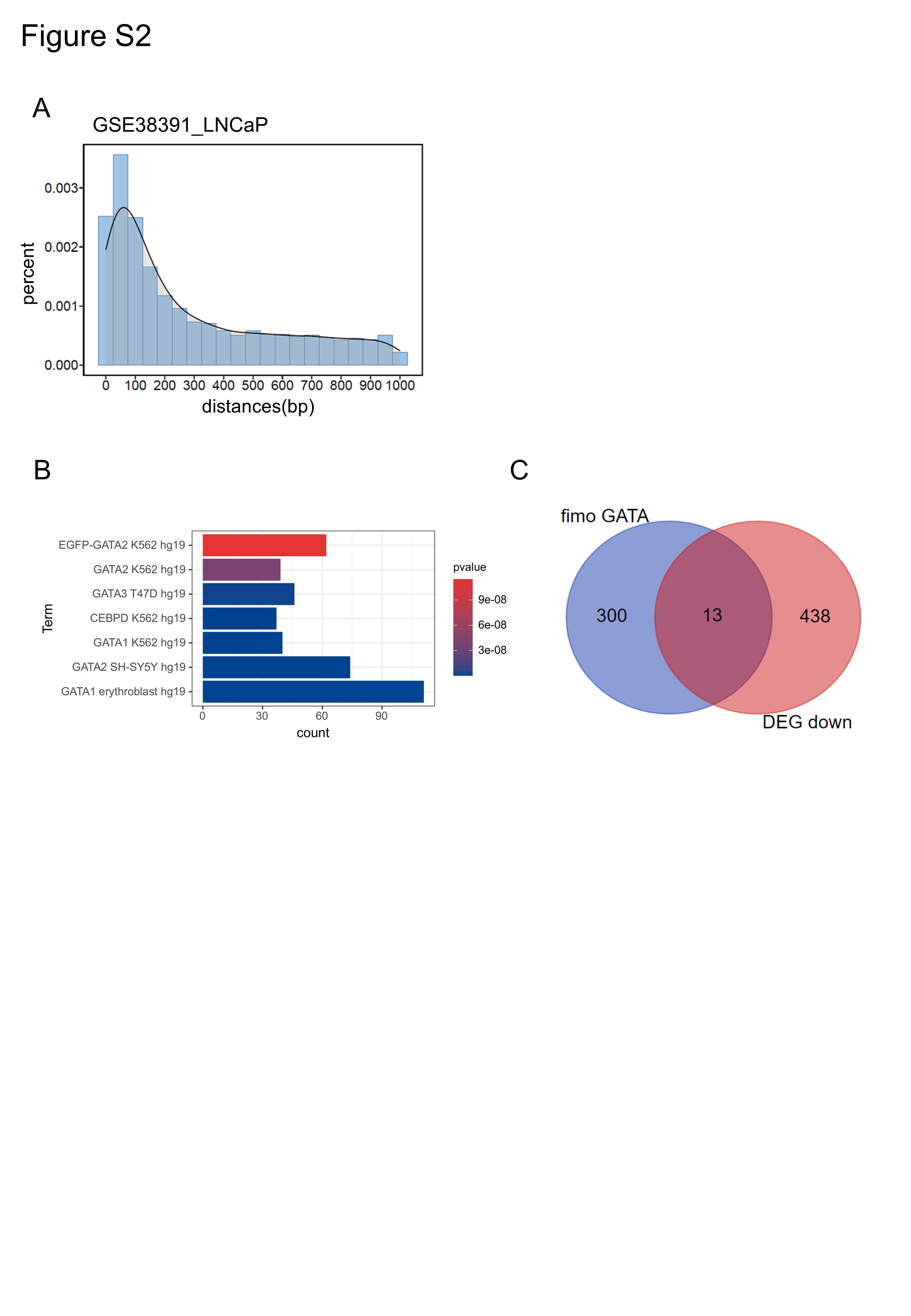

Supplement: Supplementary Figure 2 — Prediction of GATA2 targets. (A) Distribution of distances between GATA2 peaks and the nearest TSS of a gene in the GATA2 ChIP-seq from LNCaP (GSE38391). Only peaks within the promoter region (−1000 to +1000 bp) of at least one protein coding gene were included. (B) The enrichment results of 313 potential GATA-targeted genes. The number of target genes in each enriched category is shown on the x-axis. The color represents the p-value. (C) Intersection of potential GATA2 target genes predicted by FIMO (left) and DEGs down-regulated upon GATA2 activation (right). [file Image_2.TIF]

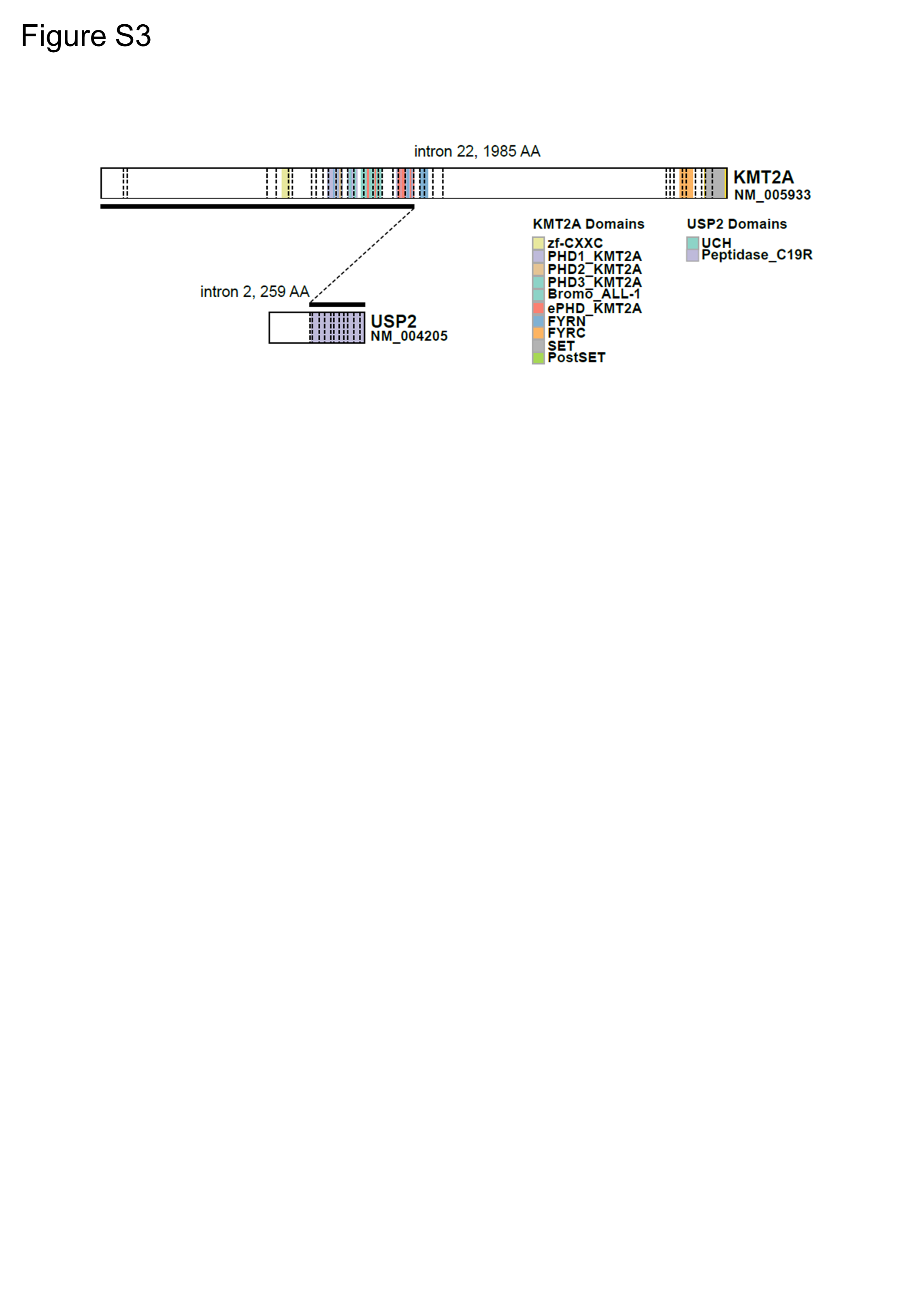

Supplement: Supplementary Figure 3 — KMT2A-USP2 fusion in MLL B-ALL patients with outlier GATA2 transcription. Protein diagrams were generated using ProteinPaint (29) (https://proteinpaint.stjude.org/). [file Image_3.TIF]

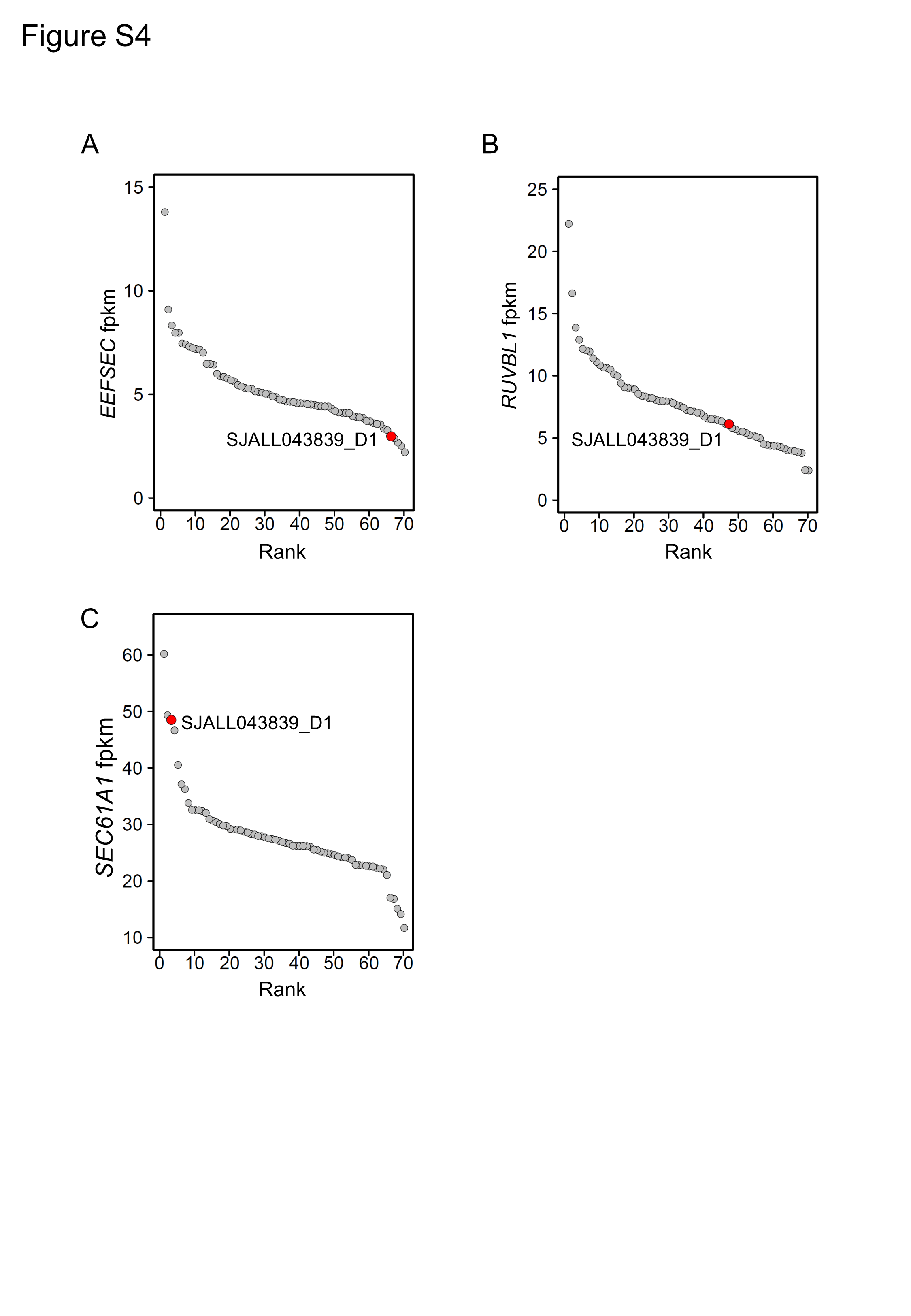

Supplement: Supplementary Figure 4 — The transcription of genes near the deletion downstream of GATA2. The expression levels of EEFSEC (A), RUBBL1 (B), and SEC61A1 (C) in B-ALL patients from the Shanghai Children's Medical Center ALL cohort are shown as scatterplots. Expression level (FPKM) is plotted on the y-axis. The case with cis-activated GATA2 (SJALL043839_D1) is highlighted in red in each panel. [file Image_4.TIF]
